# Taraxasterol prompted the anti-tumor effect in mice burden hepatocellular carcinoma by regulating T lymphocytes

**DOI:** 10.1038/s41420-022-01059-5

**Published:** 2022-05-16

**Authors:** Feng Ren, Yu Zhang, Yuanhua Qin, Jingli Shang, Yanling Wang, Pengkun Wei, Jiaming Guo, Huijie Jia, Tiesuo Zhao

**Affiliations:** 1 Basic Medical College, Xinxiang Medical University, Xinxiang, 453000 Henan PR China; 2 Henan International Joint Laboratory of Immunity and Targeted Therapy for Liver-Intestinal Tumors, Xinxiang Medical University, Xinxiang, 453000 Henan PR China; 3 Xinxiang Key Laboratory of Tumor Vaccine and Immunotherapy, Xinxiang Medical University, Xinxiang, 453000 Henan PR China; 4 Department of Pathology, Xinxiang Medical University, Xinxiang, 453000 Henan PR China; 5 Department of Immunology, Xinxiang Medical University, Xinxiang, 453000 Henan PR China

**Keywords:** Hepatocellular carcinoma, Immunotherapy

## Abstract

Hepatocellular carcinoma (HCC) is a common digestive malignant tumor with high morbidity and mortality worldwide, however, the treatment of HCC and prognosis of patients are not optimistic, finding more effective treatments are imperative. Taraxacum officinale (L.) Weber ex F.H.Wigg is a perennial herb of compositae, and our study has demonstrated that Taraxacum officinale polysaccharide has certain anti-tumor effect on HCC cells. Taraxasterol (TS) is a natural product extracted from Taraxacum officinale with strong physiological, pharmacological and biological activities, but the effect of TS on HCC is yet to be determined. Therefore, the aim of this study is to explore the effect of dandelion sterol on HCC in vivo and in vitro. The results showed that TS significantly inhibited the proliferation, induced apoptosis and blocked cell cycle in HCC cell lines HepG2 and Huh7 cells in vitro. TS inhibited the tumor growth of H22 bearing mice and the expression of Ki67 in vivo. More importantly, TS regulated the immunity of H22 bearing mice by elevating the ratio of CD4^+^ T cells in spleen, and increasing the number of T cell infiltration in tumor tissue. Except immunomodulation, the mechanism of tumor growth inhibition may be related to the regulation of apoptosis related proteins and IL-6/STAT3 pathway. TS significantly inhibited the growth of HCC cells both in vitro and in vivo. The study would provide a theoretical basis for the new application of TS and the adjuvant treatment of malignant tumor with traditional Chinese medicine.

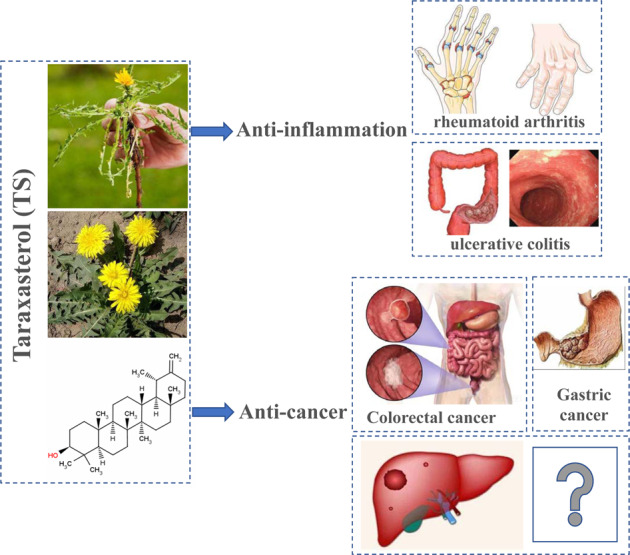

## Introduction

As a highly common malignant tumor of digestive tract, hepatocellular carcinoma ranks second of cancer deaths in the world in 2020. Even with the continuous progress of various treatment methods, the 5-year survival rate of liver cancer is only 20% [1]. Therefore, the research on the treatment of hepatocellular carcinoma has always been concerned.

Taraxacum officinale (L.) Weber ex F.H.Wigg has long been traditionally used as a kind of Chinese herbal medicine for disorders of the liver, breast and gallbladder as well as hepatitis and digestive diseases [2–6]. However, the components of Taraxacum officinale are complex, so it is very important to find the specific components of Taraxacum officinale. Taraxasterol (TS) is a natural product extracted from Taraxacum officinale with strong physiological [7], pharmacological and biological activities [8]. Researches have demonstrated that it has anti-inflammation, anti-cancer and other pharmacological activities, while the effect of TS on proliferation of the HCC cells is yet to be determined. It is easily available, wide occurrence in plants, stable properties and anti-tumor activity, making it an attractive target for drug development. Although some studies have shown that TS delayed the progression of mice with rheumatoid arthritis by inhibiting inflammatory response [7], however, its anti-tumor immune response is not clear.

Along with advances in immunology, more and more attention has been paid to the role of anti-liver cancer by regulating the immune system [9]. As we all know, the body’s immune system is suppressed in the patients with liver cancer, which leads to immune escape of tumor cells and accelerates tumor progression. Indeed, several preclinical and clinical data support this hypothesis showing that immunotherapy and even more their combination may be a good alternative candidate for the treatment of HCC patients [10, 11]. As a very important adaptive immune cell, T lymphocytes play a very important role in anti-tumor. Studies have shown that the number of T cell infiltration in tumor tissue is closely related to the prognosis of patients [12, 13]. Therefore, T cell-associated immunotherapy lights up the hope for the improvement of complementary approach to conventional HCC treatments. So far, many traditional Chinese medicines have been proved to have certain role in regulating immune function [14, 15]. Taraxacum officinale polysaccharide can be developed as a new immune enhancer because of its good immune regulation ability [16]. However, its effect on T cells needs to be further studied. In particular, the effect of sterol, a specific component of polysaccharide, on T cells is also not clear.

Therefore, in this study, the effect of TS on the proliferation of HCC cells was explored in vivo and in vitro, and its regulatory effect on T cells was further clarified. The study would provide a theoretical basis for the new application of TS and the development of Taraxacum officinale resources.

## Results

### TS significantly inhibited the proliferation of hepatoma cells

**Fig. 1:**
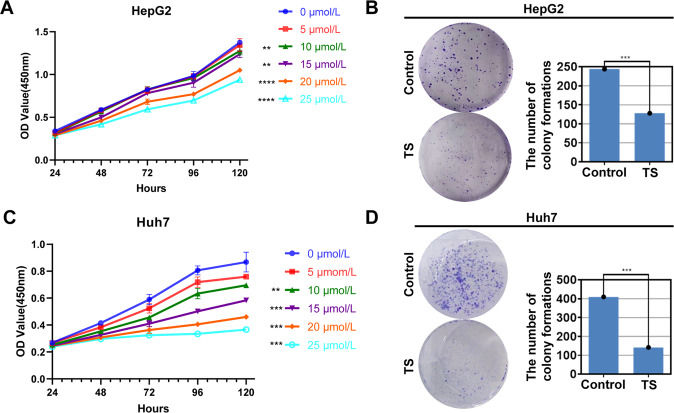
TS significantly inhibited the proliferation of hepatoma cells in vitro. **A** The effect of different concentrations of TS (0, 5, 10, 15, 20 and 25 μmol/L) on the viability of HepG2 during 24 to 120 h via CCK-8. **B** The effect of TS (15 μmol/L) on colony forming ability of HepG2 measured by cell clone formation experiment. **C** The effect of TS with different concentrations of 0, 5, 10, 15, 20 and 25 μmol/L on the viability of Huh7 from 24 to 120 h through CCK-8. **D** The effect of TS (15 μmol/L) on colony forming ability of Huh7 detected by cell clone formation experiment. Results were obtained from experiments carried out in triplicate from at least three independent experiments. Data are presented as the mean ± SD (*n* = 5). *p < 0.05 vs. the Control group; **p < 0.01 vs. the Control group; ***p < 0.001 vs. the Control group.

Firstly, we detected the effects of the TS at different concentrations (5, 10, 15, 20 and 25 μmol/L) on the growth of two hepatoma cell lines HepG2 and Huh7. The results showed that when concentration was 10 and 15 μmol/L, TS suppressed the proliferation of HepG2 cells only at 120 h, while when the concentration was more than 20 μmol/L, it markedly inhibited the proliferation of HepG2 cells at 48, 72, 96 and 120 h (Fig. 1A). The results of cell cloning experiment are consistent with the above results (Fig. 1B). In addition, the TS also inhibited the proliferation of Huh7 cells at 72, 96 and 120 h when the concentration was more than 5 μmol/L, while when the concentration reached 10 μmol/L, it extremely inhibited the proliferation of Huh7 cells for more than 48 h incubation (Fig. 1C). The cell cloning experiment shows similar results (Fig. 1D).

### Effects of TS on apoptosis and cell cycle of HepG2 and Huh7 cells

**Fig. 2:**
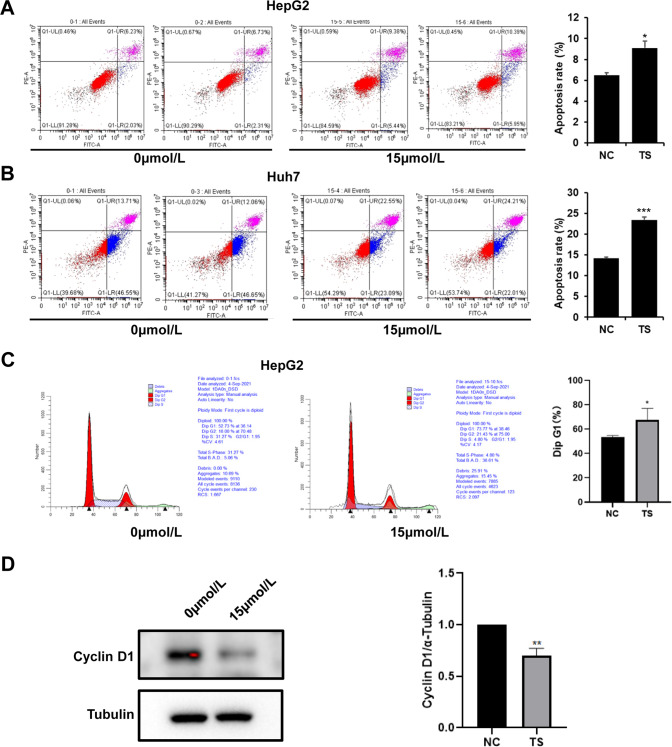
TS induced apoptosis and blocked cell cycle of hepatoma cells in vitro. The effect of TS with 15 μmol/L on apoptotic rate of HepG2 (**A**) and Huh7 (**B**) after 72 h using annexin V/PI double staining. **C** The effects of TS with 15 μmol/L on the cell cycle of HepG2 through propidium iodide staining. **D** The expression of Cyclin D1 after treated with TS by WB assay and the statistical analysis. Results were obtained from experiments carried out in triplicate from at least three independent experiments. Data are presented as the mean ± SD (*n* = 5). *p < 0.05 vs. the Control group; **p < 0.01 vs. the Control group; ***p < 0.001 vs. the Control group.

In order to detect the effect of TS on cell apoptosis and cell cycle of HepG2 and/or Huh7 cells, the Annexin V-FITC-PI apoptosis double staining and the cell cycle changes were performed by flow cytometry. The results demonstrated that the apoptosis rate of HepG2 hepatoma cells in the control group was 6.48%, however, after treatment with TS (15 μmol/L) for 72 h, the apoptosis rate of HepG2 increased to 9.095% (Fig. 2A); while the apoptosis rate of Huh7 hepatoma cells in the control group was 14.17%, and after treatment with TS (15 μmol/L) for 72 h, the apoptosis rate of Huh7 hepatoma cells increased to 23.4% (Fig. 2B). The results indicated that TS could induce apoptosis of human hepatoma cells. Moreover, the results from cell cycle showed that compared with the control group, the proportion of cells in G1 phase was increased after treat with TS, while that in G2 phase decreased was in TS group (Fig. 2C). Besides, the expression of cell cycle relative protein Cyclin D1 was also detected using WB assay, and the results showed that TS with the concentration of 15 μmol/l also markedly suppressed the expression of Cyclin D1 as compared with that in the control group (Fig. 2D, E), indicating that treatment with TS could induce G1 phase arrest of HepG2 cells.

### Effect of TS on tumor growth of tumor bearing mice

**Fig. 3:**
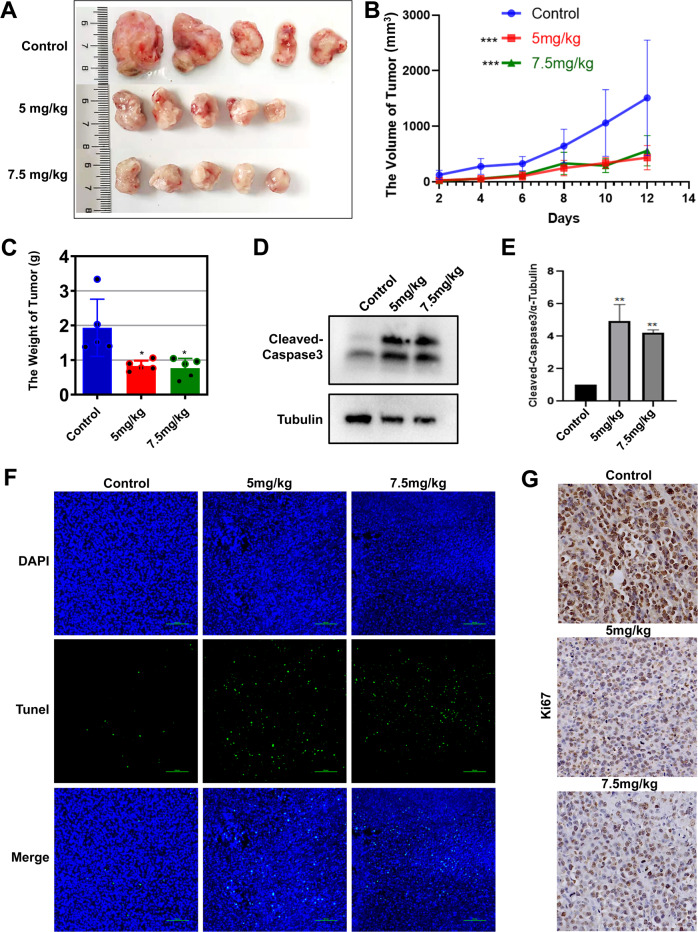
TS inhibited tumor growth of H22 tumor bearing mice in vivo. **A** The image of tumor from H22 tumor bearing mice. **B** Tumor weight of H22 tumor bearing mice. **C** Tumor volume changes of H22 tumor bearing mice. **D** The expression of cleaved Caspase-3 after treated with TS by WB assay in vivo. **E** The statistical analysis for the WB assay in **D**. **F** The apoptotic cells in tumor tissue after treatment by TUNEL. **G** Ki-67 immunohistochemistry of the tumor sections in H22 tumor bearing mice (40×). Results are from independent duplicate or triplicate experiments and presented as means ± SD. Number of mice per group are seven (*n* = 5). *p < 0.05 vs. the Control group; **p < 0.01 vs. the Control group; ***p < 0.001 vs. the Control group.

Next, we examined the effect of TS on tumor growth of H22 hepatoma-bearing mice. The results showed that TS at the doses of 5 and 7.5 mg/kg notably suppressed the tumor growth of the tumor bearing mice (Fig. 3A–C). As shown in Fig. 3D–F, the expression of apoptosis related protein cleaved caspase-3 was significantly elevated while the apoptotic cells in tumor tissues were markedly reduced after treatment with TS at the concentration of 5 or 7.5 mg/kg, which is similar to the results in vitro. Moreover, cell proliferation was measured by staining for the proliferation markers Ki-67. Immunohistochemistry for Ki-67 illustrated inhibited staining of proliferate unclears in tumor tissues after treated by TS (Fig. 3G), indicating that TS can effectively inhibit the proliferation of tumor cells.

### Effect of TS on the ratio of T cells in spleen and T cell infiltration in tumor tissue of hepatoma-bearing mice

**Fig. 4:**
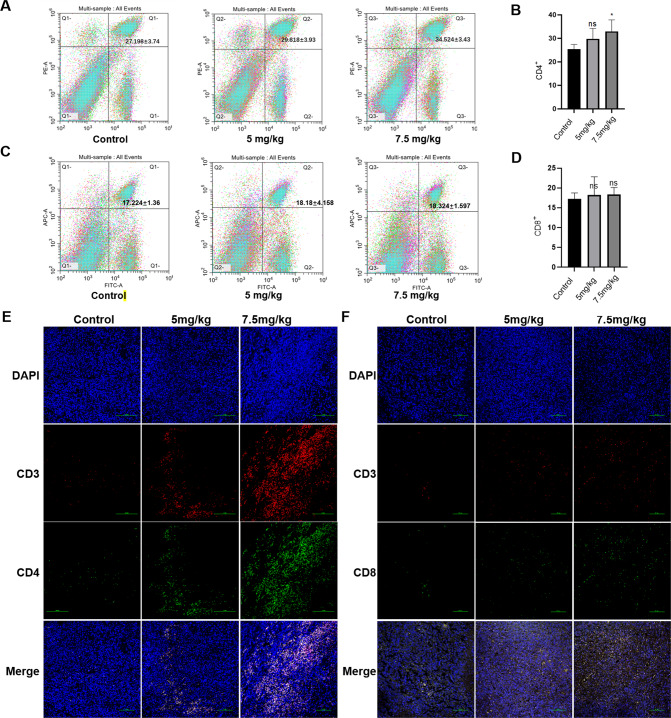
TS promoted the immune regulation of H22 tumor bearing mice. **A** The proportion of CD4^+^ T cells in the spleen of tumor bearing mice were detected by flow cytometry. **B** The analysis of the results from **A**. **C** The proportion of CD8^+^ T cells in the spleen of tumor bearing mice were detected by flow cytometry. **D** The analysis of the results from **C**. The infiltration of CD4^+^ (**E**) /CD8^+^ (**F**) T cells in tumor tissue sections were detected by immunofluorescence (20×). Results are from independent duplicate or triplicate experiments and presented as means ± SD. Number of mice per group are seven (*n* = 7). *p < 0.05 vs. the Control group; **p < 0.01 vs. the Control group; ***p < 0.001 vs. the Control group.

Many studies have shown that traditional Chinese medicine could improve the immune response. So, we next investigated the ratio of T cells in the spleen of tumor bearing mice. The results showed that TS significantly increased the ratio of CD4^+^ T cells in the spleen after treatment with a dose of 7.5 mg/kg (Fig. 4A, B), but had no significant effect on the ratio of CD8^+^ T cells (Fig. 4C, D). We further examined the effect of TS on T cell infiltration in tumor tissues of hepatoma-bearing mice. The results showed that TS with the dosage of 5 mg/kg not only increased the infiltration of CD4^+^ T cells in tumor tissue (Fig. 4E), but also increased the infiltration of CD8^+^ T cells (Fig. 4F), which is more obvious in 7.5 mg/kg group.

### Effect of TS on the expression of apoptosis related proteins and IL-6/STAT3 pathway related proteins in HCC cells

**Fig. 5:**
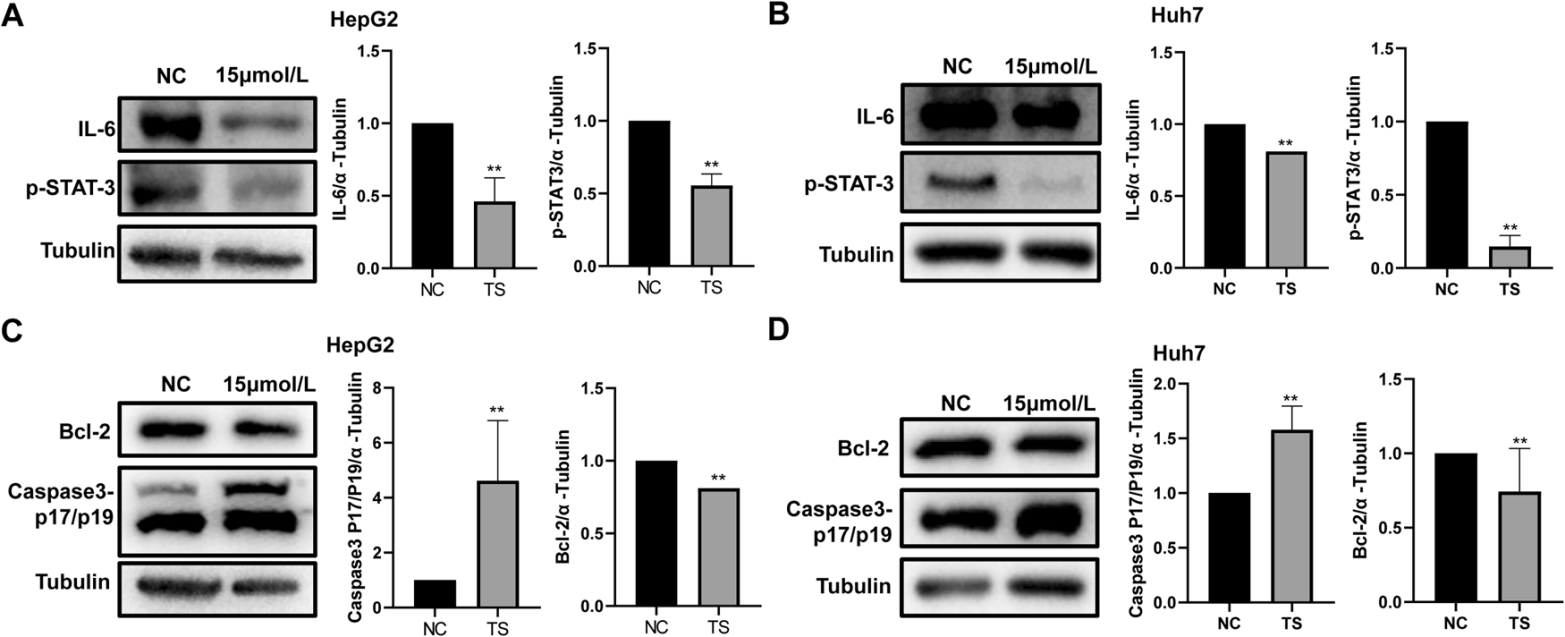
TS affected the expression of apoptosis related proteins and IL-6/STAT3 pathway related proteins in HepG2 and Huh7 cells. The expression of IL-6 and p-STAT-3 after treatment by TS (15 μmol/l) in HepG2 cells (**A**) and Huh7 cells (**B**).The expression of apoptosis related proteins (Bcl-2 and cleaved Caspase-3) after treatment by TS in HepG2 cells (**C**) and Huh7 cells (**D**). Results were obtained from experiments carried out in triplicate from at least three independent experiments. Data are presented as the mean ± SD (*n* = 5). *p < 0.05 vs. the Control group; **p < 0.01 vs. the Control group; ***p < 0.001 vs. the Control group.

In order to explore other mechanism related to the tumor inhibition by TS. We further detected several proteins expression using WB. The results showed that the translation of both oncogene STAT3 and its upstream gene IL-6 were downregulated after TS treatment (Fig. 5A, B). Additionally, the apoptotic protein marker Bcl-2 was inhibited while cleaved caspase-3 was increased (Fig. 5C, D). These results indicated that TS affected the expression of apoptosis related proteins and IL-6/STAT3 pathway related proteins in HCC related cell lines.

## Discussion

Hepatocellular carcinoma is the most common primary malignancy of the liver in adults and the main cause of cancer-related death worldwide with few efficacious therapeutic options for this deadly disease [17]. This study explored the therapeutic effect of TS on hepatocellular carcinoma. The results showed that TS is capable of inhibiting tumor cell proliferation, inhibiting the growth of tumor and improving the anti-tumor immunity of tumor bearing mice.

Cancer progression is known to involve the degree of proliferation. Taraxacum officinale root extract (TRE) has demonstrated therapeutic anti-tumor activity in different cancer cell models. It was showed that as TRE potently inhibited proliferation and migration and expedited apoptosis in gastric carcinoma cells [18]. Another study illustrated that TRE affected the proliferation by activating a multiplicity of death signaling pathways in colorectal cancer without toxicity to non-cancerous cells [19].

Our previous studies also found that DP inhibited the proliferation of HCC in vitro and in vivo [20]. In order to further clarify the role of specific components in TS, we detected the effect of TS on the proliferation of HCC cells. The results showed that it significantly inhibited the proliferation of HCC cells, which was related to the fact that sterols could inhibit the cell cycle of tumor cells. Cell proliferation specific antigen Ki67 is an important protein for the detection of cell proliferation activity [21, 22], which is often detected by immunohistochemistry. Besides, proliferating cell nuclear antigen and micro chromosome maintenance proteins are also the basic proliferation markers commonly used to evaluate the growth fraction of cell populations [23]. Our results illustrated that TS effectively inhibited HCC cell proliferation by inhibiting the expression of Ki67. These results provide experimental evidence for the development of anti-tumor drugs based on Taraxacum officinale extract.

At present, HCC has been considered an immunogenic tumor [24]. Therefore, immunotherapeutic approaches might be a more appropriate therapeutic strategy. It was confirmed that immune response and hepatocellular carcinoma progression are closely related. The role of the immune system in the control of cancer initiation and progression has been demonstrated in recent decades [25]. It was found that the anti-tumor immune response was inhibited in hepatocellular carcinoma patients, leading to immune escape. T lymphocyte, as an important immune cell, is highly important in immune response and anti-tumor response [26].

**Fig. 6:**
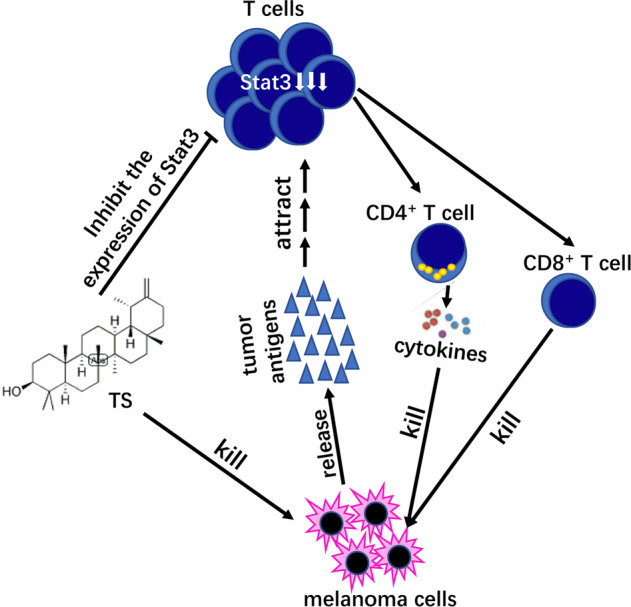
Hypothesis of the mechanism about TS anti HCC. The high expression of STAT3 will not only significantly promote tumor progression, but also mediate tumor immune escape, and the results further confirmed that TS could significantly inhibit the expression of STAT3 protein in tumor tissues. Therefore, we hypotheses that the anti-tumor mechanism of TS is as follows: firstly, TS causes the apoptosis of tumor cells, which would enable tumor cells to release more tumor antigens, chemotactic a large number of CD4^+^ and CD8^+^ T lymphocytes to reach tumor tissues and play an anti-tumor role. In addition, TS could inhibit the expression of STAT3 protein in T cells and further enhance the anti-tumor effect of T cells, and finally improve the anti-tumor immune response of tumor bearing mice.

The traditional medicine has been used for years in treatment of cancer patients. It may not only attenuate the severity of tumor symptoms and improve the quality of life, but also control the size of tumor and prolong the survival of patients. It is known that many traditional Chinese medicines inhibit tumor growth by affecting the immune system [27]. Some traditional Chinese medicines serve an anti-tumor role in immunosuppressive tumor microenvironment through upregulating immune response [28]. Tumor-infiltrating lymphocytes, which can recognize tumor cells and provoke an anti-tumor response plays an active role in the survival of patients with hepatocellular carcinoma [29]. There are also reports the ratios of tumor‐infiltrating CD4^+^/CD8^+^ are associated with the survival of the patients with HCC [30]. As effector cytotoxic T lymphocytes, CD8^+^ T cells can recognize cancer antigens presented by major histocompatibility complex molecules and dissolve tumor cells by releasing granzyme and perforin after activation. CD4^+^ T cells can enhance the function of tumor specific CD8^+^ T cells by secreting interleukin-2, which can effectively promote tumor regression [31]. Our results showed that TS not only effectively increased the ratio of CD4^+^ T cells in the spleen of tumor bearing mice, but also elevated the infiltration of CD4^+^ and CD8^+^ T cells in tumor tissues. It was confirmed that TS played an anti-tumor role by regulating T cells, though this effect is even more pronounced for the regulation of CD4^+^ T cells. The mechanism may be related to the expression of STAT3 (Fig. 6).

## Materials and methods

### Cell Counting Kit-8 analysis

Human HCC cell lines (HepG2 and Huh7) were provided by Stem Cell Bank, Chinese Academy of Sciences. TS was purchased from Chengdu Ruifensi Biotechnology Co., Ltd, China (purity ≥99%, and endotoxin free) [32]. Huh7 or HepG2 cells were prepared into 96-well plates with a density of 2 × 10^3^ cells/well and incubated for 14–16 h at 37 °C with the condition of 5% CO_2_. Then TS with the different concentrations of 0, 5, 10, 15, 20 and 25 μmol/l were added, respectively. After co-cultured for 24, 48, 72, 96 or 120 h, CCK-8 was added to each well and incubated for another 2 h. Finally, the OD value at the wavelength of 450 nm was detected by an enzyme-labeling instrument (Molecular Devices, USA). The changes of cell activity after administration for 24, 48, 72, 96 or 120 h were measured and plotted.

### Cell clone formation experiment

The cells in logarithmic growth stage were seeded into the 6-well plates, with 8 × 10^3^ cells/well (Huh7) and 2 × 10^3^ cells/well (HepG2). After 24 h, TS was added at concentrations of 0, 5, 10, 15, 20 and 25 μmol/l. The cells were observed every day, and the culture medium was changed every 2 days. When the cells grew into clusters, the culture medium was removed, and each well was washed twice with PBS and sucked out with a pipette. Then, 1 ml 4% (wt/vol) paraformaldehyde was added into each well, which was fixed for 30 min at room temperature and then removed. Subsequently, 1 ml crystal violet was added into each well and stained for 15 min. The 6-well plate was washed with ultrapure water, and then images were captured and saved. The plate was inverted and a piece of transparent film with a grid was superimposed. The number of clones with >10 cells was counted under the microscope (Nikon, Tokyo, Japan). Finally, the clone formation rate was calculated: Clone formation rate = (clones/inoculated cells) × 100%.

### Establishment of subcutaneous tumor model in mice

Mouse HCC cell line H22 was obtained from China Center for Type Culture Collection. Kunming mice (female, 6–8 weeks) were purchased from Experimental animal center of Xinxiang Medical University. The mice were fed in pathogen-free conditions, housed under a 12 h light/dark cycle at a temperature of 25 ± 2 °C. H22 cells was inoculated subcutaneously into Kunming mice. Mice were randomly divided into three groups: control group, treatment with 5 mg/kg TS group and treatment with 7.5 mg/kg TS group, with seven mice in each group. When the tumor grew to a sufficient size (about 100 mm^3^) for 5–7 days, the mice were given 0.1 ml PBS or different concentrations of TS per day via gavage for 14 days. The tumor volume of mice was measured every other day until it reached to 1500 mm^3^. The mice were killed, the tumors were removed, weighed, and photographed. Tumors from the mice were fixed overnight and embedded in paraffin. All animal studies were performed according to protocols approved by the Ethics Committee of Xinxiang Medical University.

### Apoptosis analysis

HepG2 and Huh7 cells were cultured and treated with different concentrations of TS (0 and 15 μmol/l). After 72 h, the cells (2 × 10^6^) were collected and mixed with 100 μL Annexin V binding buffer. The cells were added with 5 ul Annexin V-FITC and 5 ul PI Solution for 15 min and then were added with 400 ul 1× Annexin V Binding Solution. The ratio of apoptosis was analyzed by the flow cytometry (Cytoflex, Beckman).

### Cell cycle detection

HepG2 cells were cultured and treated with different concentrations of TS (0 and 15 μmol/l) for 72 h. The cells (5 × 10^5^ − 1 × 10^6^) were fixed with 75% ethanol at 4 °C overnight. Subsequently, cells were then washed with PBS and labeled with Propidium iodide staining solution and added with RNase A. Cell cycle assays were performed with the flow cytometry (Cytoflex, Beckman) and analyzed with Modfit Software (Topsham, ME).

### Western blot

HepG2 cells and Huh7 cells were treated with TS for 72 h. The protein was then extracted with RIPA lysis buffer (Solarbio life sciences, Beijing, China) and the concentration was determined using a bicinchoninic acid protein assay. For tumor tissue, 0.1 g of tissue were weighed and grinded, 600 ul RIPA lysate was added on ice for 30 min, and then centrifuged (8000r for 10 min) at 4 °C. The supernatant was collected, the loading buffer (Beyotime Institute of Biotechnology, Shanghai, China) was added, and the protein lysis sample was boiled for 13 min at 95 °C. The protein samples were separated by 12 and 8% sodium dodecyl sulfate-polyacrylamide gel electrophoresis and transferred to polyvinylidene fluoride membranes (Millipore, Billerica, MA, USA). The membranes were blocked with 5% non-fat milk for 2 h at room temperature and then incubated with the following primary antibodies of α-Tubulin (1:1000; Beyotime; AF0001), Caspase-3 P17/P19 (1:1000; Proteintech; 19677-1-AP), Cleaved Caspase-3 (1:1000, CST; 9664), IL-6 (1:1000, Abacm; ab258341), Bcl-2 (1:1000; proteintech; 12789-1-AP), Cyclin D1 (1:1000; Proteintech; 26939-1-AP) and p-STAT-3 (1:1000; CST; 9145) overnight at 4 °C. The membrane was then incubated with Goat Anti-Rabbit IgG antibody (1:3000, Cwbio; CW0103S). Specific immune complexes were visualized using enhanced chemiluminescence (Beyotime Institute of Biotechnology) and semi-quantified with the software of multifunctional chemiluminescence imaging system (Viliber, France).

### Immunohistochemistry

Tumor tissues were fixed in 4% formalin for 24 h, embedded in paraffin, and sectioned into 5 μm thick sections. The sections were deparaffinized and dehydrated in a series of xylene and alcohol washes. Then, antigen retrieval was performed by heating the tissue sections in a microwave for 10 min in a citrate solution (10 mmol/l; pH 6.0). The tissues were then blocked with 1% (wt/vol) BSA (Gibco; Thermo Fisher Scientific, Inc.) at room temperature for 15 min and incubated with monoclonal antibodies directed against Ki67 (1:2000; Proteintech Group, Inc., Chicago, IL, USA; 23709-1-AP) overnight at 4 °C. The sections were rinsed with PBS for 5 min and blocked with HRP-conjugated immunoglobulin G secondary antibody (1:1000; ZSGB-BIO, Beijing, China) for 30 min at room temperature. The slices were colored by DAB for 5 min at room temperature, counterstained with hematoxylin for 1 min, differentiated by hydrochloric ethanol, and finally sealed with neutral resin glue, scanned and photographed under light microscope.

### Terminal deoxynucleotidyl transferase (TdT)‐mediated dUTP nickend labeling (TUNEL)

Tumor tissues were fixed in 4% formalin for 24 h, embedded in paraffin, and sectioned into 5 μm thick sections. The apoptosis of cells in tumor tissues is detected by TUNEL assay kit (Beyotime Institute of Biotechnology, Shanghai, China) according the instruction. Firstly, the TUNEL detection solution is dropwise added on the surface of tumor sections and incubated in dark at 37 °C for 60 min. Then the sections are washed using PBS for 10 min and three times. Finally, the sections are dried and sealed with the solution of anti-fluorescence quenching, and observed the image using the fluorescence microscope.

### Flow cytometry

The spleen was isolated under sterile conditions and grounded with ground glass. The suspension is sucked out, filtered with a filter screen (200 mesh, 70UM), and then centrifuged. The erythrocyte lysate was added for 2 min, which was centrifuged and then was adjusted to 1 × 10^6^/ml. The antibodies CD3/CD4/CD8 were added respectively and incubated on ice in dark for 30 min. In total, 1640 of 200 ul was added to resuspended cells and centrifuged. The supernatant was discarded and the cells were washed with 300 ul precooled PBS. Each sample was filtered with filter trap EP with light avoid. The sample was tested with flow cytometry immediately.

### Immunofluorescence

Tumor tissues were fixed in 4% formalin for 24 h, embedded in paraffin, and sectioned into 5 μm thick sections. The antigen was repaired by microwave and the endogenous catalase activity was eliminated by 3% catalase. The sections were blocked with 10% goat serum and incubated with primary anti mouse anti-CD3 (1:100)/mouse anti-CD4 (1:150)/rabbit anti-CD8 (1:600) at 4 °C overnight. The slices were washed with PBS for three times, and then incubated with fluorescent secondary antibody (1:200) at room temperature for 30 min. The slices were washed three times with PBS and then incubated with DAPI at room temperature for 5 min. The slices were washed with PBS for three times, and then sealed with anti-fluorescence attenuation sealing solution. Photos were taken using fluorescence microscope, and five visual fields were selected for each slice.

### Statistical analysis

Data are designated as the means ± SD of three independent experiments. One-way ANOVA was performed to test the difference among the different groups. Each experiment was repeated at least three times with each data point done in duplicate. The binding data were analyzed by GraphPad Prism software. *p* < 0.05 were considered to indicate a statistically significant difference.

## Supplementary information

Original Data File

Original Data File

Original Data File

Original Data File

## Data Availability

The data used to support the findings of this study are available from the corresponding author upon request.
