# Supplementary figures and images for: Taraxasterol prompted the anti-tumor effect in mice burden hepatocellular carcinoma by regulating T lymphocytes
Source: Cell Death Discov. 2022 May 16;8:264. doi: 10.1038/s41420-022-01059-5 (PMC9110731; doi:10.1038/s41420-022-01059-5)

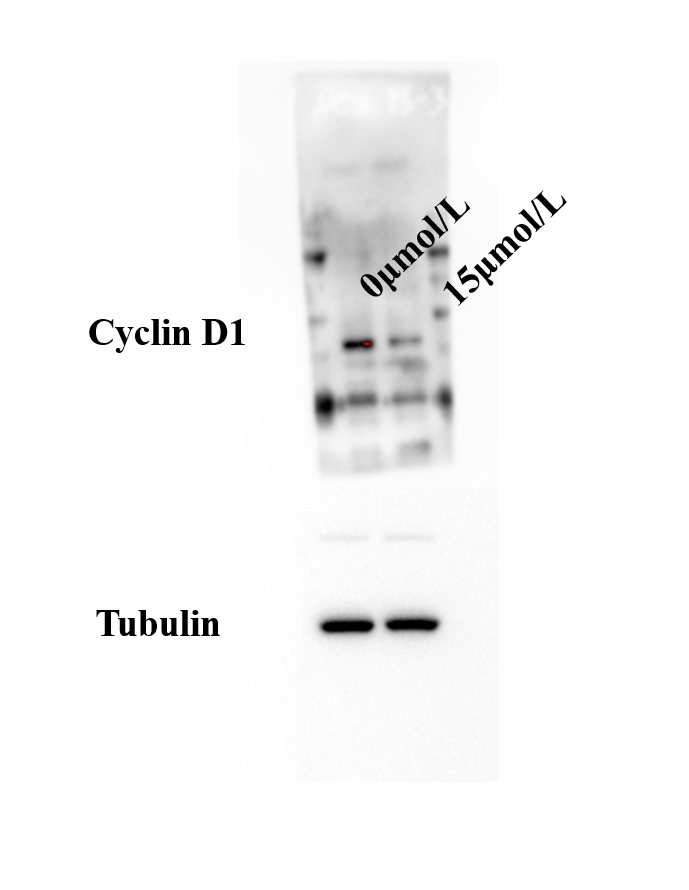

Supplement: Supplementary file 1 — Original Data File [file 41420_2022_1059_MOESM1_ESM.tif]

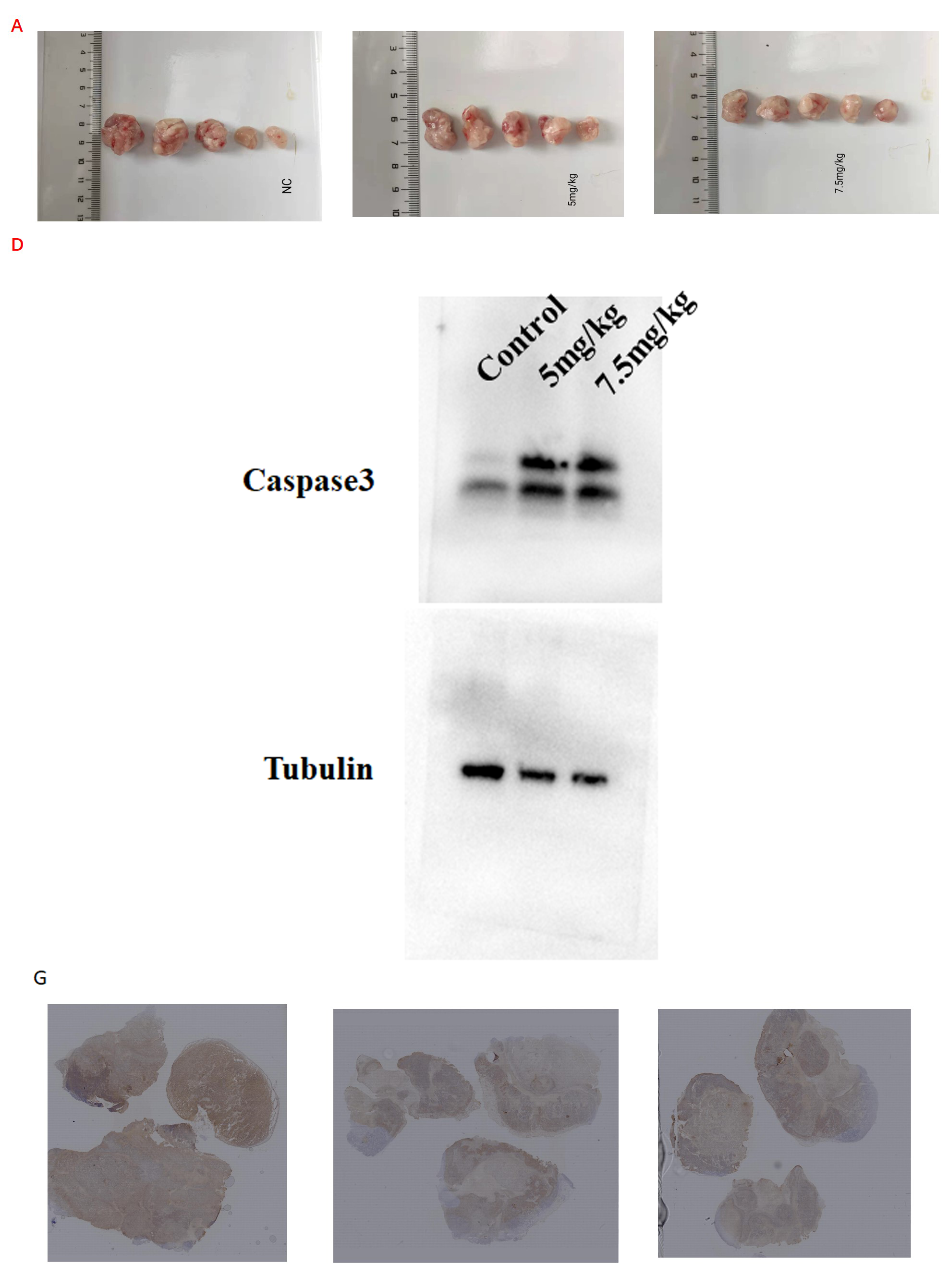

Supplement: Supplementary file 2 — Original Data File [file 41420_2022_1059_MOESM2_ESM.tif]

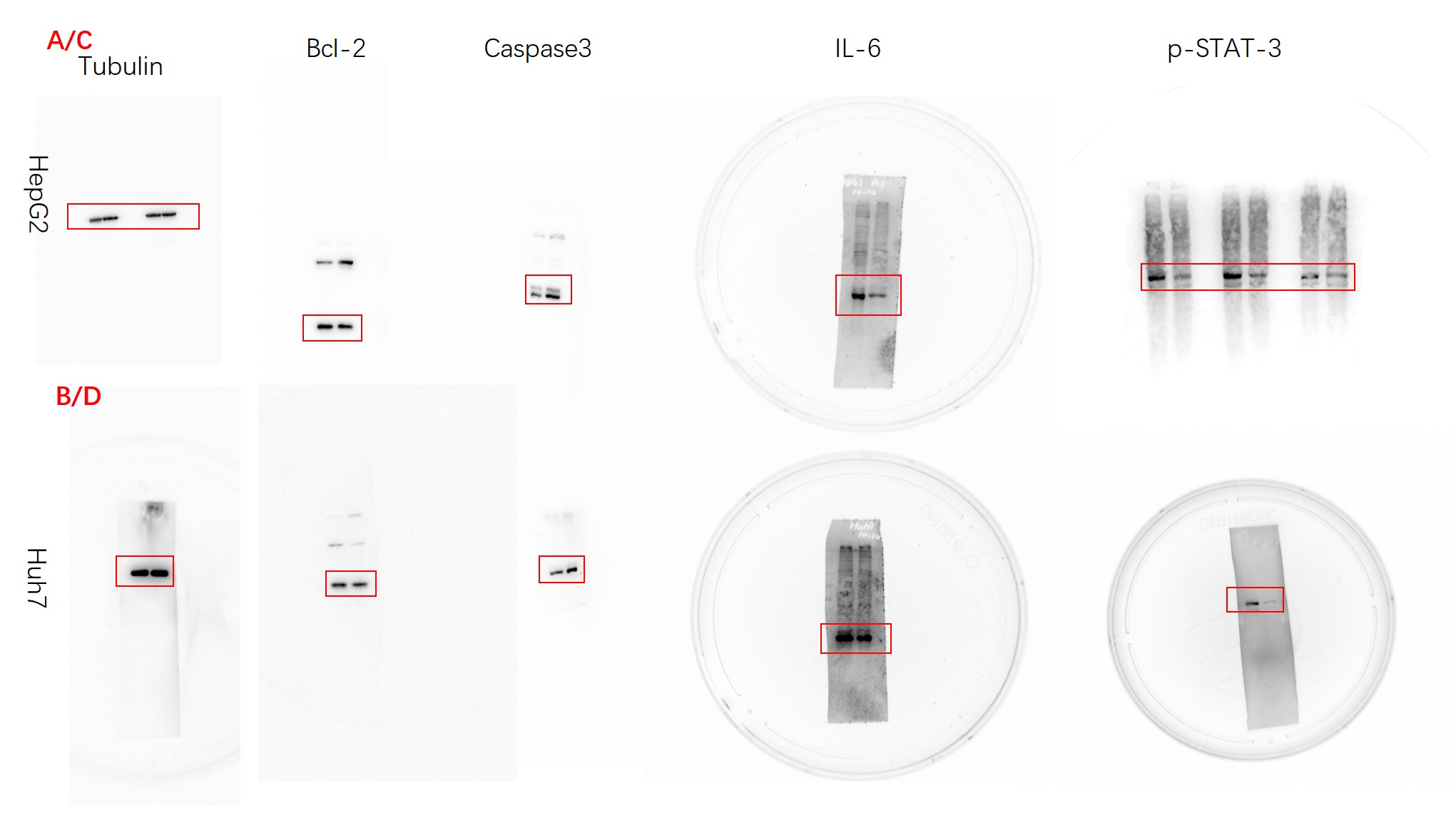

Supplement: Supplementary file 3 — Original Data File [file 41420_2022_1059_MOESM3_ESM.jpg]

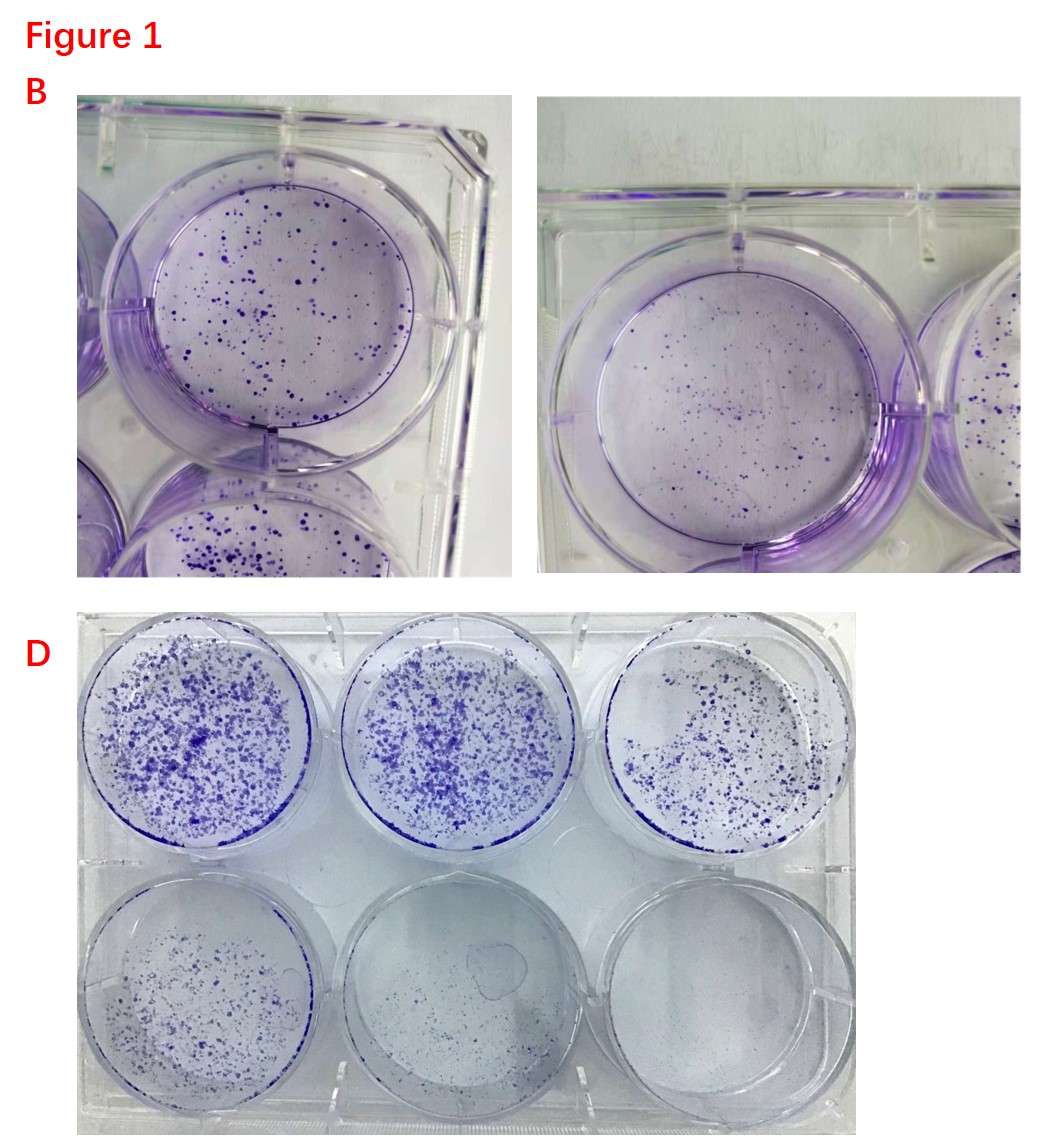

Supplement: Supplementary file 4 — Original Data File [file 41420_2022_1059_MOESM4_ESM.jpg]
